# Pulmonary surfactant protein B carried by HDL predicts incident CVD in patients with type 1 diabetes

**DOI:** 10.1016/j.jlr.2022.100196

**Published:** 2022-03-14

**Authors:** Baohai Shao, Janet K. Snell-Bergeon, Laura L. Pyle, Katie E. Thomas, Ian H. de Boer, Vishal Kothari, Jere Segrest, William S. Davidson, Karin E. Bornfeldt, Jay W. Heinecke

**Affiliations:** 1 Department of Medicine, University of Washington, Seattle, WA, USA; 2 Barbara Davis Center for Diabetes, University of Colorado Denver, Aurora, CO, USA; 3 Department of Pediatrics, School of Medicine, University of Colorado Anschutz Medical Campus, Aurora, CO, USA; 4 Department of Medicine, Vanderbilt University School of Medicine, Nashville, TN, USA; 5 Center for Lipid and Arteriosclerosis Science, Department of Pathology and Laboratory Medicine, University of Cincinnati, Cincinnati, OH, USA

**Keywords:** SFTPB, HDL proteomics, incident CVD, MS, parallel reaction monitoring, CACTI, case-cohort design, atherosclerosis, plasma fractionation, smoking status, AMBP, alpha-1-microglobulin/bikunin precursor, APO, apolipoprotein, APOA1, apolipoprotein A1, C4BPA, C4b binding protein alpha chain, CACTI, Coronary Artery Calcification in Type 1 Diabetes study, CEC, cholesterol efflux capacity, CST3, cystatin C gene 3, FDR, false discovery rate, HbA1c, hemoglobin A1c, HR, hazard ratio, ICAM-1, intracellular adhesion molecule-1, IGF1, insulin-like growth factor 1, PON, paraoxonase, PRM, parallel reaction monitoring, SFTPB, pulmonary surfactant protein B, T1DM, type 1 diabetes mellitus, VCAM-1, vascular cell adhesion molecule-1

## Abstract

Atherosclerotic CVD is the major cause of death in patients with type 1 diabetes mellitus (T1DM). Alterations in the HDL proteome have been shown to associate with prevalent CVD in T1DM. We therefore sought to determine which proteins carried by HDL might predict incident CVD in patients with T1DM. Using targeted MS/MS, we quantified 50 proteins in HDL from 181 T1DM subjects enrolled in the prospective Coronary Artery Calcification in Type 1 Diabetes study. We used Cox proportional regression analysis and a case-cohort design to test associations of HDL proteins with incident CVD (myocardial infarction, coronary artery bypass grafting, angioplasty, or death from coronary heart disease). We found that only one HDL protein—SFTPB (pulmonary surfactant protein B)—predicted incident CVD in all the models tested. In a fully adjusted model that controlled for lipids and other risk factors, the hazard ratio was 2.17 per SD increase of SFTPB (95% confidence interval, 1.12–4.21, *P* = 0.022). In addition, plasma fractionation demonstrated that SFTPB is nearly entirely bound to HDL. Although previous studies have shown that high plasma levels of SFTPB associate with prevalent atherosclerosis only in smokers, we found that SFTPB predicted incident CVD in T1DM independently of smoking status and a wide range of confounding factors, including HDL-C, LDL-C, and triglyceride levels. Because SFTPB is almost entirely bound to plasma HDL, our observations support the proposal that SFTPB carried by HDL is a marker—and perhaps mediator—of CVD risk in patients with T1DM.

Diabetes is a major public health problem in the US population (1). Type 1 diabetes mellitus (T1DM) accounts for ∼5% of all cases of diabetes. Both type 1 and type 2 diabetic patients have a markedly increased risk of atherosclerotic CVD. Moreover, CVD is the major cause of morbidity and mortality in people with T1DM (2, 3). However, traditional lipid and nonlipid cardiovascular risk factors do not appear to completely explain the extent and severity of cardiovascular events in T1DM (4). Thus, it is important to identify new factors that predict CVD risk in this population.

Clinical and epidemiological studies show a robust and inverse association of HDL-C levels with CVD risk (5, 6). Although dyslipidemia associates independently with CVD in T1DM (7), HDL-C levels are not consistently low and are even elevated in patients with T1DM despite increased CVD risk (8, 9, 10). Moreover, several studies indicate that pharmacological elevation of HDL-C did not reduce CVD risk in statin-treated patients (11, 12). Collectively, these observations indicate that HDL-C levels do not necessarily reflect the cardioprotective effects of HDL, indicating the need for HDL metrics of CVD risk that are distinct from HDL-C.

HDL carries a wide array of proteins linked to lipoprotein metabolism, inflammation, protease inhibition, and complement regulation (13), suggesting that its protein cargo may influence the cardioprotective properties of HDL independently of traditional CVD risk factors. MS-based proteomics demonstrate that ∼200 proteins are detected in HDL (14). Low levels of paraoxonase (PON) 3, one of the HDL proteins, associate with subclinical atherosclerosis (15). We recently showed that a low level of the antiatherosclerotic HDL protein PON1 associates with both albuminuria and prevalent atherosclerosis as quantified by coronary artery calcification in patients with T1DM (16). Conversely, high levels of PON1 in HDL associated with long-term protection against vascular complications in patients with T1DM (17).

It is unclear whether specific HDL proteins predict incident CVD events in patients with T1DM. This information could be important for understanding the mechanisms underlying accelerated atherosclerosis in T1DM and for identifying risk factors that predict CVD in this population. In the current study, we used serum samples from subjects enrolled in the Coronary Artery Calcification in Type 1 Diabetes (CACTI) study, a prospective evaluation of diabetic complications, to determine whether the HDL proteome predicts incident CVD. We found that levels of one HDL protein—pulmonary surfactant protein B (SFTPB)—predicted incident CVD. Our observations indicate that the protein cargo of HDL is a marker of CVD risk in patients with T1DM in this cohort.

## Materials and methods

### Study design and subjects

We used 181 serum samples from patients with T1DM in the CACTI study (18) for our analyses. The CACTI study prospectively evaluated 1,416 subjects (652 patients with T1DM and 764 nondiabetic subjects), 19–56 years of age, for a wide range of complications of diabetes (19). At enrollment, subjects were diagnosed with T1DM before the age of 30 years or had autoantibodies and a clinical course consistent with T1DM. All CACTI subjects with T1DM began insulin treatment within 1 year of diagnosis of diabetes. The mean duration of diabetes was 23 years at enrollment; the minimum was 4 years. After an overnight fast, blood was collected and centrifuged, and plasma and serum were stored at −80°C for future analysis.

The study design has been reported (18). To determine the levels of HDL proteins in T1DM control subjects relative to those of T1DM subjects with incident CVD, we used the subjects in the randomly selected cohort without CVD (134 of 145 subjects) and all 47 cases with incident CVD, respectively. To determine whether HDL proteins associated with incident CVD risk, we used a case-cohort design and Cox hazard analysis. The cases were all 47 subjects with CVD, and the cohort was 145 randomly selected subjects (134 control subjects and 11 case subjects). This approach enabled us to perform analyses of the randomly selected cohort that were representative of the full population (20, 21).

All CACTI subjects with T1DM who were free of known CVD when samples were collected were eligible for the study. The cases were all 47 subjects who had an available sample and an adjudicated CVD event. The cohort consisted of 145 subjects randomly selected from all subjects; it included 11 subjects who subsequently suffered incident CVD. CVD was defined as myocardial infarction, coronary artery bypass grafting, angioplasty, or death from coronary heart disease. Subjects were followed for CVD for an average of 16 years after baseline samples were collected. All subjects provided informed consent. The study was approved by the Colorado Combined Institutional Review Board. Samples were deidentified, and MS/MS analyses were blinded.

### Materials and reagents

Unless otherwise specified, all reagents were obtained from Sigma-Aldrich (St. Louis, MO). Water and acetonitrile for MS analyses were Optima LC/MS grade (Fisher Scientific, Pittsburg, PA). Formic acid was from EMD Millipore (Billerica, MA). ^15^N-labeled apolipoprotein (APO) A1 ([^15^N]APOA1, ^15^N enrichment 99+%) was produced with a bacterial expression system (22). Stable isotope-labeled peptides mimicking trypsin proteolysis products of SFTPB (VVPLVAGGI(C)Q(C)LAERˆ and SPTGEWLPRˆ, (C) = Cys-carbamidomethylation, Rˆ = arginine [^13^C_6_, ^15^N_4_]) were purchased from New England Peptide, Inc. (Gardner, MA). Their purity was confirmed by MS and MS/MS analysis. Full-length pro-SFTPB recombinant protein was from ABNOVA (Taipei, Taiwan).

### Laboratory measurements

Total cholesterol and triglyceride levels were measured using standard enzymatic methods. HDL-C was quantified by dextran sulfate precipitation, and LDL-C was calculated using the Friedewald formula (23). Hemoglobin A1c (HbA1c) was quantified by high-performance liquid chromatography.

### HDL isolation

HDL (density 1.063–1.210 g/ml) was isolated by sequential ultracentrifugation from rapidly thawed serum (never thawed), using buffers supplemented with 100 μM diethylenetriaminepentaacetic acid and a protease inhibitor cocktail (Sigma, St. Louis, MO) (16). Protein concentration was determined using the Lowry assay (Bio-Rad), with albumin as the standard (24).

### Digestion of HDL proteins

Following the addition of freshly prepared methionine (5 mM final concentration) in 20% acetonitrile and 100 mM NH_4_HCO_3_, 5 μg of HDL protein was reduced with dithiothreitol and then alkylated with iodoacetamide. After adding 0.2 μg of isotope-labeled [^15^N]APOA1, we incubated HDL overnight at 37°C with 20:1 (w/w, protein/enzyme) sequencing grade-modified trypsin. Digestion was halted by acidifying the reaction mixture (pH 2–3) with trifluoroacetic acid, and the samples were dried and stored at −80°C until MS analysis.

### Protein nomenclature

Protein symbols (all capital letters, not italicized) are based on gene symbols recommended by the Human Genome Organization Gene Nomenclature Committee (25).

### LC-ESI-MS/MS analysis of HDL proteins by parallel reaction monitoring

We used targeted proteomics with isotope-dilution parallel reaction monitoring (PRM) to quantify HDL proteins (26, 27). Briefly, LC-ESI-MS/MS analyses were performed in the positive-ion mode with an ultrahigh-resolution accurate mass Orbitrap Fusion Lumos Tribrid mass spectrometer (Thermo Fisher Scientific, San Jose, CA) coupled to a nanoACQUITY UPLC (Waters, Milford, MA). A multistep gradient of 0.1% formic acid in water (solvent A) and 0.1% formic acid in acetonitrile (solvent B) was used for the separation. HDL peptide digests (equivalent to 0.2 μg of protein) were desalted on a C-18 trap column (0.1 × 40 mm) at a flow rate of 2.5 μl/min for 8 min. They were then separated at a flow rate of 0.5 μl/min using a C-18 analytical column (0.1 × 200 mm). Both the trap and analytical columns were packed in house with Magic C-18 reverse-phase resin (5 μm; 100 Å; Michrom Bioresources, Inc). The columns were kept at room temperature, and the peptides were separated using a multistep gradient as follows: 1–7% solvent B for 1 min, 7–25% solvent B for 24 min, 25–35% solvent B for 6 min, and 35–80% solvent B for 5 min. The column was subsequently washed for 3 min in 80% solvent B and reequilibrated in 99% solvent A for 12 min. The mass spectrometer was operated in the data-independent acquisition PRM mode.

Results of our previous studies (16, 27, 28) and a preliminary shotgun analysis of HDL isolated from 40 CACTI subjects were used to select 50 HDL proteins for relative quantification. Initially, at least two peptides from one protein were tested by PRM test runs, and finally, two peptides were selected for 35 proteins and one peptide for 15 proteins for quantification (supplemental Table S1). The peptides selected for one protein were unique to that protein. Because oxidation of methionine residues might affect the quantification, we avoided methionine-containing peptides.

### Quantifying HDL proteins with [^15^N]APOA1

The relative levels of proteins in HDL were quantified by PRM with isotope dilution, using [^15^N]APOA1 as the internal standard (27). The PRM data were analyzed with Skyline (version 21.1.0.278, University of Washington, Seattle, WA), an open source program (29). An equal amount of [^15^N]APOA1 was added to HDL isolated from each subject prior to digestion as an internal standard. Based on the journal guidance from the *Molecular & Cellular Proteomics*, our targeted PRM analyses belong to tier level 3. The peak areas of all the transitions of a peptide detected by PRM analysis were summed to get the total peak area for the peptide, but the transitions with interferences were deleted. To normalize the peak area of a peptide, the total peak area of all selected transitions of the peptide was divided by the peak area of one peptide (DYVSQFEGSALGK) from ^15^N-APOA1, and the ratios were used for quantification. To calculate the relative levels of peptides between the control and CVD groups, we set the average ratio of the peptide in control subjects (the subjects in the cohort group excluding the 11 CVD cases) to an arbitrary value of one. To obtain the relative level of a protein in HDL, if two peptides were quantified, the relative levels of the two peptides from each protein were averaged.

### Isolation and digestion of HDL, LDL, VLDL, and lipoprotein-free plasma

HDL (density 1.063–1.21 g/ml), LDL (1.006–1.063 g/ml), VLDL (0.95–1.006 g/ml), and lipoprotein-free plasma (>1.21 g/ml) were isolated by sequential ultracentrifugation from rapidly thawed plasma from 10 T1DM subjects in the cohort (30). Lipoproteins and lipoprotein-free plasma were dialyzed against 20 mM potassium phosphate (pH 7.0) containing 100 μM diethylenetriaminepentaacetic acid (31).

Lipoprotein-associated proteins were digested as described previously for HDL. To digest plasma proteins, 5 μl plasma was diluted in 245 μl of 25 mM NH_4_HCO_3_. A 10 μl aliquot of diluted plasma (equivalent to 0.2 μl of plasma and 14 μg of protein) was digested as reported previously. (18) Full-length pro-SFTPB (381 amino acids, recombinant protein with glutathione-*S*-transferase tag at the N terminus [Abnova, Taipei, Taiwan]) was used as an external standard. Peptide digests were analyzed for SFTPB, APOA1, APOA4, APOB, and APOE by isotope-dilution PRM. SFTPB was quantified using two stable isotope-labeled peptides (VVPLVAGGI(C)Q(C)LAERˆ and SPTGEWLPRˆ, (C) = Cys-CAM [carbamidomethylation], Rˆ = arginine [^13^C_6_, ^15^N_4_]) that were predicted trypsin proteolysis products of SFTPB. Levels of peptides from other proteins were normalized to [^15^N]APOA1. HDL proteins were given an arbitrary value of 100; levels of proteins in other lipoproteins and lipoprotein-free plasma were expressed as percentages of their levels in HDL.

### Cholesterol efflux capacity

The ability of serum HDL (precipitated by polyethylene glycol) to promote cholesterol excretion by murine J774 macrophages and ABCA1-transfected BHK cells was assessed as previously described (31, 32). Briefly, the macrophage cholesterol efflux capacity (CEC) of serum HDL was determined using J774 macrophages radiolabeled with [^3^H]-cholesterol and incubated with cyclic AMP to induce ABCA1. ABCA1 CEC was quantified in BHK cells with inducible human ABCA1. The cells were incubated without or with mifepristone so they expressed virtually no ABCA1 or high levels of the transporter (33).

### Anti-inflammatory activity of SFTPB-enriched HDL

HDL was isolated from pooled control plasma by sequential ultracentrifugation as described previously. HDL (1 mg/ml protein, 250 μg of HDL) was incubated with 16.5 μg recombinant human SFTPB (0.066 mg/ml) in PBS containing 10 mM of reduced glutathione at 37°C for 2 h and then reisolated by ultracentrifugation (density, 1.063–1.210 g/ml). About 50% of HDL was recovered by ultracentrifugation of the incubation mixture. The levels of SFTPB in reisolated SFTPB-enriched HDL were measured by PRM analysis using isotope-labeled peptides as internal standards as described previously.

The anti-inflammatory activities of control HDL and SFTPB-enriched HDL were quantified using human coronary artery endothelial cells (34). Briefly, endothelial cells (from a 39-year-old woman; lot number: 01354; Frederick, MD) were treated with control HDL or SFTPB-enriched HDL (50 μg/ml) for 18 h, followed by stimulation with human recombinant TNF-α (20 ng/ml, PeproTech, Inc, Rocky Hill, NJ) for 6 h. mRNA levels of vascular cell adhesion molecule-1 (VCAM-1) and intracellular adhesion molecule-1 (ICAM-1) were then quantified by real-time PCR (34).

### Statistical analysis

Categorical variables are presented as percentages. Continuous variables are expressed as means and SDs (baseline characteristics) or as medians and interquartile ranges for variables with skewed distributions (triglyceride levels). Continuous variables were compared using the two-tailed unpaired Student’s *t-*test for normally distributed data or the Mann-Whitney nonparametric test for non-normally distributed data. Normality of distribution was assessed with the Shapiro-Wilk test. Categorical variables were compared using the Chi-square test.

To account for multiple comparison testing, *P* values obtained from the Mann-Whitney test were corrected using the method of Benjamini and Hochberg (35, 36, 37). This step-up method controls the false discovery rate (FDR) and assumes a non-negative correlation. A corrected *P* value threshold is calculated based on an FDR of 10%. Only proteins with a corrected *P* value (*q* value) <0.10 were considered significantly different. Univariate and multivariate models were built with Cox proportional hazard ratio (HR) regression. HRs, 95% confidence intervals, and *P* values were calculated using a case-cohort design and the “cch” function in R with default Prentice weighting. HRs are reported per one SD increment of HDL proteins or other variables. Two-sided *P* values <0.05 were considered statistically significant. All statistical analyses were performed with SPSS software (version 19; Chicago, IL) or the R 3.6.2 statistical computing environment (http://www.R-project.org).

## Results

### Clinical characteristics

The clinical characteristics of the 134 control subjects without incident CVD in the randomly selected subcohort and all 47 cases with incident CVD from the CACTI study (18) are given in Table 1. The cases and controls had similar BMIs, similar levels of HDL-C, LDL-C, and C-reactive protein, and were on similar doses of insulin. The percentages of males and females were also similar. In contrast, the CVD subjects were significantly older and had longer diabetes duration than the control subjects in the randomly selected cohort. They also had higher systolic and diastolic blood pressure and higher levels of HbA1c, total cholesterol, and triglycerides. The CVD subjects also had lower estimated glomerular filtration rate levels and contained more current smokers and more subjects treated with statins, angiotensin-converting enzyme inhibitors, and antihypertension medication.

**Table 1:** Clinical characteristics of CACTI subjects

Characteristic	Cohort controls without incident CVD	All cases with incident CVD in CACTI	*P*
Number of subjects	134	47	
Age (years)	35 (29–44)	45 (38–50)	<0.0001^a^
Gender (female)	73 (54.5)	21 (44.7)	0.25
DM duration (years)	22 (16–29)	32 (24–37)	<0.0001^b^
Systolic BP (mm Hg)	116 (108–127)	126 (114–136)	0.03^b^
Diastolic BP (mm Hg)	77 (72–82)	80 (74–85)	0.02^b^
Body mass index (kg/m^2^)	25.9 (22.8–28.0)	26.7 (23.5–30.7)	0.24^a^
Cholesterol (mg/dl)	166 (146–193)	183 (163–209)	0.0023^a^
Triglycerides (mg/dl)	73 (55–99)	99 (72–132)	0.00024^a^
HDL-C (mg/dl)	54 (46–64)	55 (41–68)	0.74^a^
LDL-C (mg/dl)	95 (77–114)	97 (84–131)	0.080^a^
HbA1c (%)	7.4 (6.7–8.3)	8.2 (7.6–8.8)	<0.0001^a^
C-reactive protein (μg/ml)	1.13 (0.85–1.90)	1.44 (0.94–2.56)	0.15^a^
Current smoker	14 (10.4)	14 (29.8)	0.0016
eGFR (ml/min/1.73 m^2^)	112 (93–123)	93 (61–106)	<0.0001^a^
Insulin dose (U/kg/d)	0.59 (0.48–0.72)	0.60 (0.43–0.73)	0.69^a^
Medications			
ACE inhibitor	45 (33.6)	27 (57.4)	0.0040
ARB	6 (4.5)	4 (3.0)	0.30
Antihypertensive	53 (39.6)	35 (74.5)	<0.0001
Statin	18 (13.4)	24 (51.1)	<0.0001

ACE, angiotensin-converting enzyme; ARB, angiotensin receptor blocker; BP, blood pressure; DM, diabetes mellitus; eGFR, Chronic Kidney disease-Epidemiology Collaboration formula for estimated glomerular filtration rate; Statin, HMG-CoA reductase inhibitor.

Control subjects were all subjects without incident CVD in the randomly selected cohort, and cases were all subjects with incident CVD in CACTI (18). Values are median (interquartile range) for continuous covariates and *N* (%) for categorical covariates. *P* values are from a Mann-Whitney *U* test for abnormally distributed variables.

a Student’s *t-*test for normally distributed variables.

b or a Pearson Chi-square test for categorical variables.

### Isotope-dilution PRM analysis demonstrated that five HDL proteins differed in relative abundance between control subjects without incident CVD and subjects with incident CVD

**Fig. 1:**
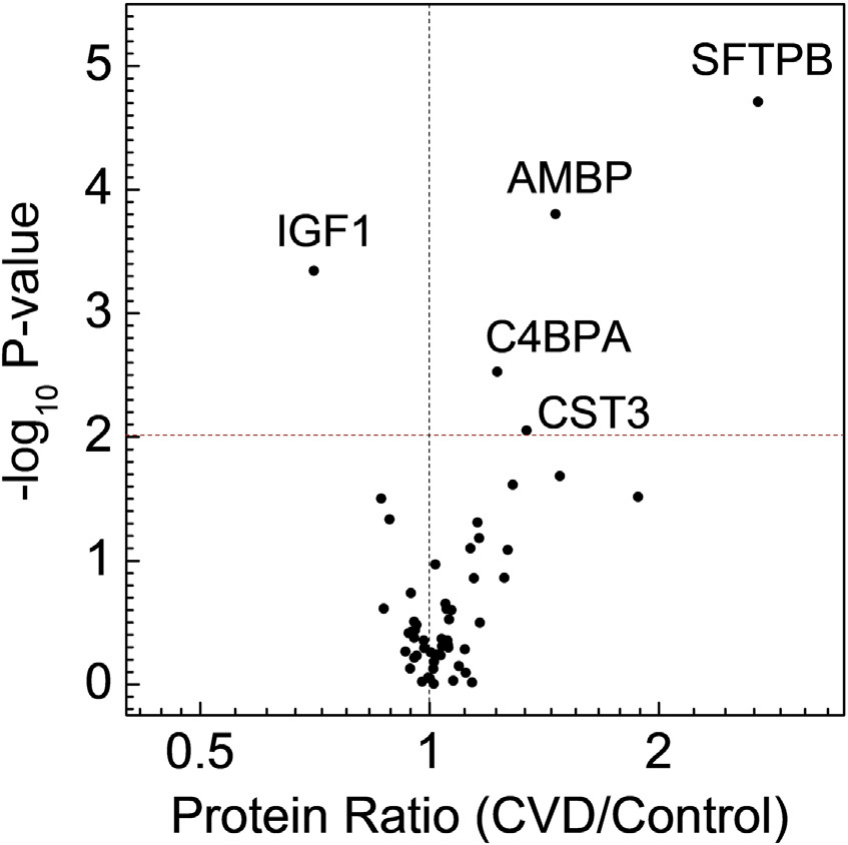
Relative HDL protein levels in patients with incident CVD subjects and control subjects. Levels of 50 proteins from 181 T1DM subjects were quantified in tryptic digests of HDL by isotope dilution and PRM. For each protein, the *P* value from the Mann-Whitney nonparametric test was plotted against the log_2_ fold mean difference in protein level between all 47 cases in CACTI and 134 control subjects in the subcohort. After controlling for multiple comparisons, proteins with *P* value <0.01 (dotted horizontal line) differed significantly in relative abundance. Proteins overexpressed in HDL of subjects with CVD have a value >1 on the *x*-axis; underexpressed proteins have a value <1.

We tested the hypothesis that the levels of one or more HDL proteins associate with incident CVD in subjects with T1DM. HDL was isolated from freshly thawed serum by ultracentrifugation, and its protein composition was quantified by isotope-dilution MS/MS with PRM (26, 27). An unadjusted analysis revealed that 11 proteins were differentially expressed in HDL isolated from the control group and from the incident CVD group (supplemental Table S2). After adjustment for multiple comparisons (Benjamini-Hochberg FDR at 10%), levels of five HDL proteins—SFTPB, insulin-like growth factor 1 (IGF1), alpha-1-microglobulin/bikunin precursor (AMBP), C4b binding protein alpha chain (C4BPA), and cystatin C gene 3 (CST3)—remained significant (*P* values <0.01) (Fig. 1). The mean IGF1 level in HDL from subjects with CVD was lower than in HDL from the control subjects (supplemental Table S2). In contrast, mean levels of the other significant proteins (SFTPB, AMBP, C4BPA, and CST3) were higher in HDL isolated from the subjects with CVD. supplemental Fig. S1 shows the comparison between the levels of SFTPB in HDL of incident CVD subjects and control subjects. It is interesting to note that the levels of SFTPB in HDL were correlated with 24 other HDL proteins that we measured (supplemental Table S3).

### Cox hazards regression identified three HDL proteins that associated with incident CVD

We next constructed Cox proportional hazards models for each of the HDL proteins using a case-cohort design and then adjusted our results for multiple comparisons by controlling the Benjamini-Hochberg FDR at 10% (FDR-adjusted *P* values or *q* values <0.10) to determine if any of the 50 HDL proteins associated with incident CVD risk (supplemental Table S4). Only proteins with a *P* value <0.008 were considered significant. In this analysis, three proteins in HDL—SFTPB, AMBP, and IGF1—were associated with incident CVD. IGF1 was not previously known to associate with HDL http://homepages.uc.edu/∼davidswm/HDLproteome.html, but we detected multiple IGF1 peptides with high confidence in the lipoprotein (supplemental Fig. S2).

**Fig. 2:**
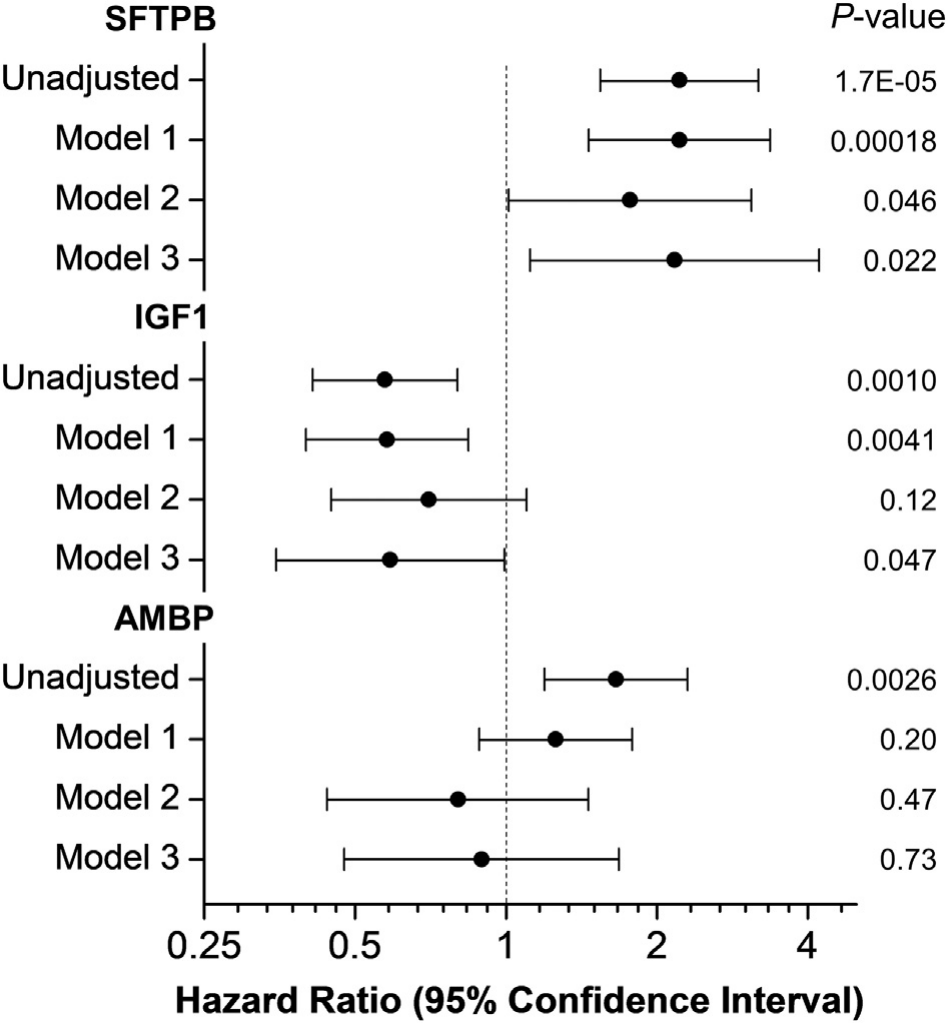
HRs of HDL proteins for incident CVD. Unadjusted and adjusted HRs, 95% confidence intervals (horizontal lines), and *P* values were from Cox proportional hazard ratio models using a case-cohort design and Prentice weighting in R. The total number of subjects was 181 (47 incident CVD subjects and 145 cohort subjects; 11 cohort subjects had incident CVD). HRs are per SD increase of levels of HDL proteins. Model 1 is a model adjusted for age, sex, and DM duration. Model 2 is model 1 further adjusted for current smoking status, levels of HbA1c and eGFR, and therapy with an ACE inhibitor and/or statin. Model 3 is model 2 further adjusted for HDL-C, LDL-C, and triglycerides. ACE, angiotensin-converting enzyme; DM, diabetes mellitus; eGFR, estimated glomerular filtration rate.

We then constructed models that adjusted for CVD risk factors that differed significantly between the control and CVD groups (Fig. 2). A model that adjusted for age, sex, and T1DM duration demonstrated that only SFTPB and IGF1 associated with incident CVD (Fig. 2, model 1). When we further adjusted the model for potential nonlipid confounders, including smoking status, HbA1c, estimated glomerular filtration rate, angiotensin-converting enzyme inhibitor therapy, and lipid-lowering therapy, only SFTPB remained strongly (HR = 1.77) and significantly (*P* = 0.046) associated with incident CVD (Fig. 2, model 2). Finally, when we further adjusted the model for HDL-C, LDL-C, and log-transformed triglyceride levels, both SFTPB and IGF1 associated with incident CVD (Fig. 2, model 3). These analyses demonstrate that high levels of SFTPB strongly (HRs = 1.8–2.2) and consistently associated with incident CVD in T1DM subjects after adjustments for a wide range of potentially confounding factors.

We previously showed that serum levels of APOC3 predicted incident CVD in CACTI (18). In the current study, levels of APOC3 in HDL also associated with incident CVD in the unadjusted model (HR = 1.40, *P* = 0.048, supplemental Table S4). However, the association lost significance after adjustments for multiple comparisons (*q* value >0.1). We also found that a model that used levels of SFTPB and APOC3 in HDL as covariates did not change the association of SFTPB with incident CVD (HR = 2.16, *P* = 0.000056). In that model, APOC3 did not associate with incident CVD (HR = 1.14, *P* = 0.49). Taken together, these results indicate that the association of SFTPB with incident CVD is independent of APOC3 levels in HDL.

### SFTPB, smoking status, and CVD risk in T1DM subjects

Multiple studies have found that smoking associates with elevated levels of circulating SFTPB and that SFTPB is a risk factor for prevalent CVD in smokers (38, 39, 40). To determine the impact of smoking status on levels of SFTPB in HDL, we first determined median levels of the protein in smokers, former smokers, and nonsmokers in the cohort group. Of the 145 subjects, 17 were current smokers, 33 were former smokers, and 95 had never smoked. The median level of SFTPB in HDL of the current smokers (3.47 arbitrary units) was significantly higher than that in HDL from the former smokers (0.68 arbitrary units) and subjects who had never smoked (0.60 arbitrary units, *P* < 0.0001 for both comparisons) (supplemental Fig. S3). The median level of SFTPB in HDL of the former smokers was not different from the subjects who had never smoked (0.68 vs. 0.60 arbitrary units, *P* = 0.98).

**Fig. 3:**
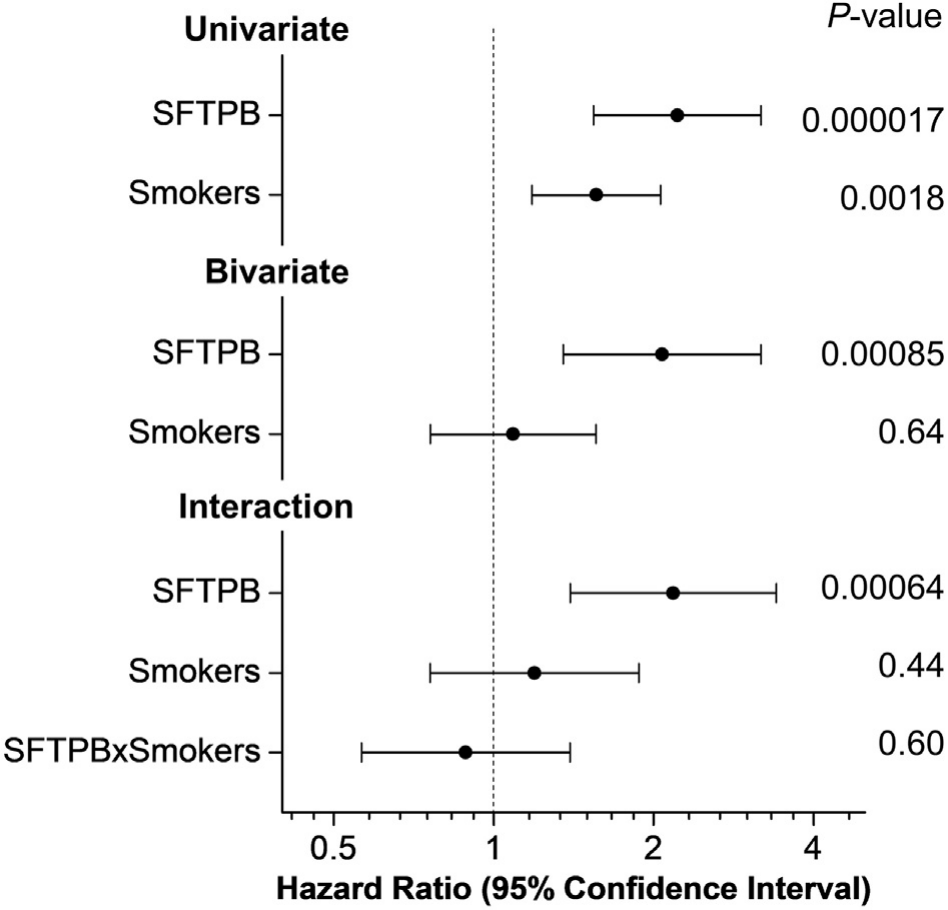
Levels of SFTPB in HDL of patients with T1DM are associated with incident CVD independent of the smoking status. HDL proteins were quantified by isotope-dilution PRM. HRs of univariate, bivariate, and interaction models, 95% confidence interval, and *P* values were obtained from Cox proportional hazard ratio models using a case-cohort design and the “cch” function in R with the default Prentice weighting. HRs are per SD increase of variables.

To further investigate the relationships among smoking status, SFTPB, and incident CVD, we repeated the Cox proportional HR regression analysis using univariate, bivariate, and an interaction term between the levels of SFTPB and current smoking status (Fig. 3). In the univariate analysis for SFTPB and current smokers, both were associated with incident CVD. However, in the bivariate (i.e., adjusted) analysis, SFTPB remained significantly associated with incident CVD. In contrast, current smokers no longer associated with incident CVD. When we further added the interaction term between the levels of SFTPB and current smokers in the model, we found no significant interaction between them. Adjusting for current smokers and adding the interaction term also did not change the strength of the associations between levels of SFTPB in HDL and incident CVD (HRs of 2.22, 2.07, and 2.18, respectively). Collectively, our observations strongly suggest that elevated levels of SFTPB in HDL are a risk factor for incident CVD in patients with T1DM and that this strong association is independent of smoking status.

### Pro-SFTPB selectively binds to HDL in plasma

SFTPB is expressed in the lung and secreted into the alveoli as a preproprotein that can be proteolytically cleaved into its mature form (41). Using MS/MS, we analyzed two isotope-labeled peptides and recombinant full-length human SFTPB protein to determine which forms of SFTPB bind to HDL in human plasma. This approach detected nine peptides in SFTPB: four in the mature protein domain, four in the propeptide domain, and one in both the mature and propeptide domains (supplemental Fig. S4). Our observations indicate that the SFTPB we quantified in HDL by MS is pro-SFTPB, which is consistent with a previous study using immunoblot analysis (42).

To analyze the distribution of SFTPB in plasma, we isolated VLDL, LDL, HDL, and lipoprotein-free plasma from 10 CACTI subjects by ultracentrifugation. Equal amounts of peptides from each digested fraction were injected into the mass spectrometer, and levels of SFTPB and apolipoproteins characteristic of each class of lipoprotein were quantified by isotope-dilution PRM. SFTPB was quantified with two stable isotope-labeled peptides based on the sequence of the human protein. The other proteins were quantified using [^15^N]APOA1.

**Fig. 4:**
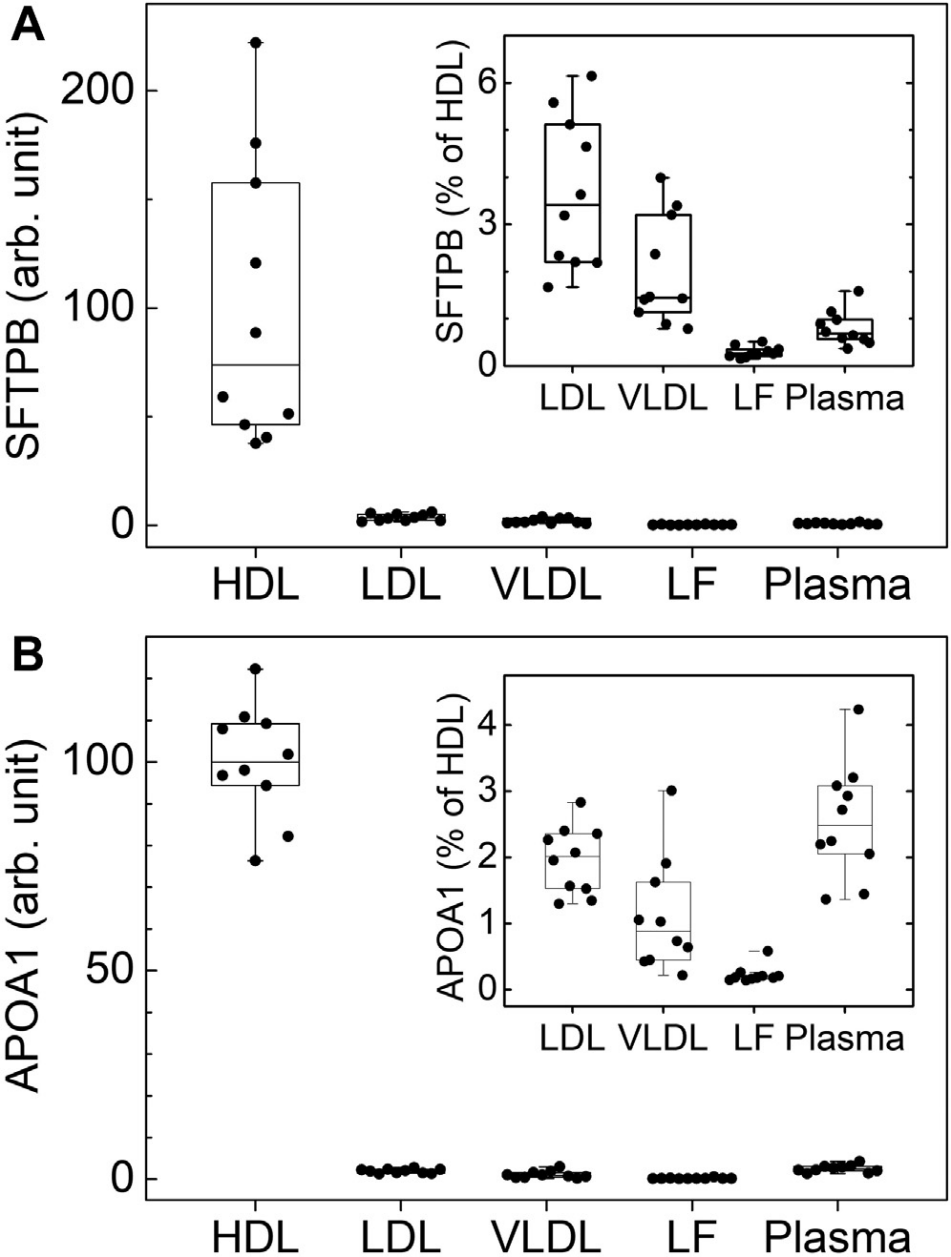
Levels of (A) SFTPB and (B) APOA1 in HDL, LDL, VLDL, and lipoprotein-free plasma. Lipoproteins and lipoprotein-free plasma were isolated by ultracentrifugation from 10 CACTI subjects. Levels of SFTPB and APOA1 were quantified by isotope-dilution PRM. SFTPB was quantified with two isotope-labeled synthetic peptides. APOA1 was quantified with ^15^N-isotope-labeled APOA1. Relative levels of SFTPB and APOA1 in HDL were defined as 100 arbitrary units. Relative levels of the two proteins in other lipoproteins and lipoprotein-free plasma are expressed as the percentage of HDL levels. The box plots show the distribution of the data of HDL proteins (median and interquartile range), and the dots represent individual data points.

Relative mean levels of SFTPB and apolipoproteins in HDL were defined as 100 arbitrary units. Levels of the proteins in other lipoproteins and lipoprotein-free plasma were expressed as percent of levels in HDL. This analysis revealed that HDL contained a much larger amount of SFTPB than LDL (only ∼3.5% of HDL levels) or VLDL (∼2% of HDL levels) (Fig. 4A, inset). Moreover, SFTPB was almost undetectable in lipoprotein-free plasma. We also found the following: *i*) HDL was greatly enriched in APOA1 (Fig. 4B, inset); *ii*) HDL, LDL, and VLDL contained similar levels of APOA4, which was mostly lipid free; *iii*) APOB was present at high levels in LDL and VLDL; and *iv*) APOE was detected primarily in VLDL (supplemental Fig. S5). These results are fully consistent with the known distributions of those proteins in isolated lipoproteins and lipoprotein-free plasma. Taken together, these observations strongly support the proposal that SFTPB is carried almost exclusively by HDL in plasma.

### SFTPB levels correlate weakly and positively with CEC

To assess the ability of serum HDL to promote cholesterol efflux, a metric that is proposed to reflect the cardioprotective functions of HDLs in humans (43, 44, 45), we used two cell models: murine J774 macrophages (macrophage CEC) and BHK cells transfected with human ABCA1 (ABCA1 CEC). Correlation analysis revealed that levels of SFTPB in HDL were significantly but weakly associated with CEC of serum HDL by J774 macrophages or by ABCA1 BHK cells (supplemental Fig. S6). Our results suggest that levels of SFTPB accounted for ∼7% of the variance in macrophage CEC and ABCA1 CEC. Moreover, the associations between levels of SFTPB in HDL and CECs were positive. Thus, modulation of CEC is unlikely to explain the association of SFTPB with incident CVD.

### Enrichment of HDL with SFTPB fails to alter the anti-inflammatory effects of HDL on endothelial cells

To determine whether SFTPB might alter the anti-inflammatory properties of HDL, we tested the ability of HDL and HDL enriched with pro-SFTPB to inhibit TNF-α-induced gene expression of two monocyte adhesion molecules (VCAM-1 and ICAM-1) in human coronary endothelial cells (34). Despite a 20-fold enrichment of HDL with pro-SFTPB, we found no differences between the levels of VCAM-1 and ICAM-1 mRNA (*P* = 0.19 and *P* = 0.89, respectively) in TNF-α-stimulated cells treated with HDL or SFTPB-enriched HDL (supplemental Fig. S7).

## Discussion

Isotope-dilution PRM analysis of 50 HDL proteins demonstrated that AMBP, C4BPA, CST3, IGF1, and SFTPB differed in relative abundance between control subjects of a randomly selected cohort and subjects with incident CVD in CACTI, a prospective study of complications in patients with T1DM. We then assessed the associations between HDL proteins and incident CVD, using Cox proportional hazards models with a case-cohort design. Only SFTPB was significantly associated with incident CVD in all the models we adjusted for potential confounders. Biochemical studies demonstrated that the proform of SFTPB was associated with HDL and that it was almost quantitatively bound to HDL in plasma. Taken together, our observations indicate that SFTPB is an HDL protein that associates with incident CVD independently of HDL-C, LDL-C, triglycerides, and a wide range of other established CVD risk factors, including smoking status, in patients with T1DM.

Previous studies have linked elevated levels of SFTPB in HDL with heart disease, diabetes, and renal disease. High concentrations of surfactant protein B (HDL) significantly associated with all-cause mortality in hemodialysis patients with type 2 diabetes mellitus (46). HDL isolated from patients with coronary heart disease or type 2 diabetes mellitus was enriched in SFTPB (47, 48). A model based on 12 HDL proteins that included SFTPB was a significant predictor of death in patients with heart failure (49).

SFTPB is synthesized almost exclusively in the lung, primarily by type 2 alveolar pneumocytes and nonciliated bronchiolar cells, which secrete the protein into the alveolar space (41). Damage to the alveolar-capillary barrier function is believed to contribute to increased leakage of pro-SFTPB from the lung (39). Smoking increases plasma levels of SFTPB likely because of alveolar inflammation and increased lung permeability (50). In the Dallas Heart Study (38), increased levels of SFTPB correlated strongly with multiple measures of smoking exposure. Importantly, in that cohort, SFTPB independently associated with prevalent abdominal aortic atherosclerosis in current smokers but not in former smokers or nonsmokers. We also found significantly elevated levels of SFTPB in HDL from current smokers compared with former smokers and nonsmokers. In contrast to previous studies, SFTPB levels associated significantly with incident CVD in Cox models after adjustment for smoking status. Importantly, in a bivariate analysis, the levels of SFTPB in HDL, but not current smoking status, significantly associated with incident CVD. There was no interaction between the levels of SFTPB and current smoking status. Our observations strongly suggest that the association of SFTPB in HDL with incident CVD and atherosclerosis in T1DM subjects is independent of smoking status.

SFTPB is an amphipathic lipid binding surfactant protein that is critical for the functioning of pulmonary surfactant, a surface-active lipoprotein complex composed of both lipids and proteins (51, 52). A key question is whether SFTPB bound to HDL directly increases CVD risk or if SFTPB bound to other lipoproteins or free in plasma are possible mediators. To address this issue, we isolated HDL, LDL, VLDL, and lipoprotein-free plasma by ultracentrifugation and quantified levels of SFTPB by isotope-dilution PRM, using isotope-labeled peptides based on the sequence of SFTPB. Essentially, all the SFTPBs were bound to HDL, strongly suggesting that HDL is its major carrier in plasma. Similar results have been found using semiquantitative immunoblot analysis (42).

A major question is why levels of SFTPB carried by HDL associate with incident CVD risk in patients with T1DM. Elevated levels of SFTPB in smokers likely reflect impaired alveolar-capillary barrier function (53). T1DM has been proposed to alter pulmonary function by unclear mechanisms (54). One possibility is that elevated levels of SFTPB in HDL are a marker for impaired capillary barrier function, perhaps because of microvascular injury, that affects both the lungs and cardiovascular system in patients with T1DM.

One mechanism might be that HDL enriched with SFTPB loses its cardioprotective functions. For example, a previous study (42) demonstrated that enrichment with SFTPB impaired the antioxidant capacity of HDL, one proposed cardioprotective function of HDL. Promotion of cholesterol efflux and inhibition of inflammation are two other proposed protective functions (38, 39, 40). However, we found that SFTPB levels accounted for only ∼7% of the variance in the CEC of subjects in our cohort. Moreover, in contrast to the hypothesis that increased CEC is cardioprotective, we found that SFTPB correlated positively with CEC. We also found no evidence that enrichment with SFTPB altered the ability of HDL to inhibit the induction of mRNAs for VCAM-1 and ICAM-1 in human endothelial cells stimulated with TNF-α. Our observations suggest that SFTPB is unlikely to promote atherosclerosis and CVD risk in T1DM subjects by impairing CEC or the anti-inflammatory effects of HDL on endothelial cells.

IGF1 associated with incident CVD in three of the four models we tested. Although this protein was not previously known to associate with HDL, we detected low levels of multiple IGF1 peptides with high confidence in the lipoprotein. However, several lines of evidence indicate that IGF1 in HDL is unlikely to be a major mediator of CVD risk in patients with T1DM. First, ∼98% of plasma IGF1 is bound to six IGF1 binding proteins, indicating that HDL is only a minor carrier (55). Second, although the interactions of IGF1 with its binding proteins are implicated in many of its biological effects, we were unable to detect known IGF1 binding proteins in HDL. Third, local production of IGF1 by endothelial cells and smooth muscle cells is implicated in the protein’s effects on arterial tissue and vascular disease (56). Finally, low levels of plasma IGF1 predict CVD in humans (57), and we found that low levels of IGF1 in HDL associate with CVD. Taken together, these observations strongly suggest that HDL is not a major carrier of IGF1 and that levels of IGF1 in HDL reflect plasma levels of the protein.

Strengths of our study include a well-characterized cohort of T1DM subjects enrolled in CACTI, quantification of HDL proteins with a well-validated proteomic approach, and stringent statistical analyses using multivariate Cox proportional hazards models. Our study also has several limitations. Despite the large number of subjects enrolled in CACTI, relatively few had incident CVD, reflecting the many advances in diabetes management over the past decade. Another limitation is that the association of SFTPB in HDL with incident CVD does not prove that SFTPB is a causal risk factor. One possibility is that enrichment of HDL with SFTPB is simply a reporter of a pulmonary dysfunction that increases the risk for cardiovascular events and mortality, perhaps by damaging the microvasculature of the lungs and artery wall (53, 54).

Although our study is likely to be relevant to the risk of CVD in patients with T1DM in the current treatment era, it will be important in future studies to confirm and extend our observations. It also will be critical to investigate the mechanisms underlying the association of SFTPB in HDL with incident CVD in T1DM.

In conclusion, we demonstrate that SFTPB in HDL predicted incident CVD in CACTI subjects who had T1DM. The association was independent of traditional CVD risk factors, including smoking status and HDL-C, LDL-C, and triglyceride levels. Importantly, because SFTPB in plasma is found almost exclusively in HDL, our observations support the proposal that SFTPB carried by HDL is a novel marker of CVD risk in patients with T1DM.

## Data availability

The data supporting this study are available in the article, the supplemental data, or available from the corresponding author upon reasonable request. The raw MS data have been deposited at the Panorama server (https://panoramaweb.org/cactihdl.url).

## Supplemental data

This article contains supplemental data.

## Conflict of interest

Jay Heinecke is named as a coinventor on patents for using oxidation and protein markers to predict the risk of CVD. All other authors declare that they have no conflicts of interest with the contents of this article.
