# Supplementary material for: Pulmonary surfactant protein B carried by HDL predicts incident CVD in patients with type 1 diabetes
Source: J Lipid Res. 2022 Mar 14;63(4):100196. doi: 10.1016/j.jlr.2022.100196 (PMC9010748; doi:10.1016/j.jlr.2022.100196)
Supplement: Supplemental Figures S1–S7 [file mmc1.pdf]

Supplemental Table 1. Peptides Monitored for Each Protein by PRM Analysis.

Proteins	Description	Peptides	Precursor (m/z)	Charge State	RT (min)
A2M	Alpha-2-macroglobulin	IAQWQSFQLEGGLK	802.925	2	29.8
A2M		LLIYAVLPTGDVIGDSAK	923.022	2	34.0
AHSG	Alpha-2-HS-glycoprotein	EHAVEGDCDFQLLK	554.259	3	23.2
AMBP	Alpha-1-Microglobulin/Bikunin Precursor	GECVPGEQEPEPILIPR	960.48	2	27.6
AMBP		TVAACNLPIVR	607.34	2	23.4
ANTXR1	Anthrax toxin receptor 1	ASEQIYYENR	636.796	2	15.2
ANTXR1		DLGAIVYCVGVK	647.347	2	29.8
ANTXR2	Anthrax toxin receptor 2	LDGLVPSYAEK	596.316	2	22.2
ANTXR2		VSPVGETYIHEGLK	510.272	3	21
^{15}N-APOA1	^{15}N-labeled Apolipoprotein A-I	DYVSQFEGSALGK	708.3160	2	25.7
^{15}N-APOA1		LHELQEK	454.2291	2	12.5
APOA1	Apolipoprotein A-I	DYVSQFEGSALGK	700.8380	2	25.7
APOA1		LHELQEK	448.7450	2	12.5
APOA2	Apolipoprotein A-II	EQLTPLIK	471.2870	2	22.6
APOA2		SPELQAEAK	486.7540	2	13.2
APOA4	Apolipoprotein A-IV	LAPLAEDVR	492.2796	2	19.9

APOA4		LGEVNTYAGDLQK	704.359	2	20
APOB	Apolipoprotein B	TEVIPPLIENR	640.864	2	27.6
APOB		VPSYTLILPSLELPVLHVPR	748.444	3	34.9
APOC1	Apolipoprotein C-I	EFGNTLEDK	526.7484	2	16.3
APOC1		EWFSETFQK	601.2798	2	25.8
APOC2	Apolipoprotein C-II	ESLSSYWESAK	643.799	2	23.5
APOC2		TAAQNLYEK	519.2670	2	14
APOC3	Apolipoprotein C-III	DYWSTVK	449.719	2	19.2
APOC3		GWVTDGFSSLK	598.8009	2	27.9
APOC4	Apolipoprotein C-IV	AWFLESK	440.7320	2	26.0
APOC4		ELLETVVNR	536.804	2	22.3
APOD	Apolipoprotein D	NILTSNNIDVK	615.8380	2	21.8
APOE	Apolipoprotein E	LAVYQAGAR	474.767	2	15.8
APOE		LGPLVEQGR	484.78	2	18.1
APOF	Apolipoprotein F	SGVQQLIQYYQDQK	849.4280	2	28.1
APOF		SGVQQLIQYYQDQK	566.621	3	28.1
APOF		SYDLDPGAGSLEI	668.8170	2	30.9

APOH		ATFGCHDGYSLDGPEEIECTK	796.004	3	22.6
APOH		CSYTEDAQCIDGTIEVPK	1043.459	2	24.5
APOL1	Apolipoprotein L-I	LNILNNNYK	553.304	2	21.8
APOM	Apolipoprotein M	WIYHLTEGSTDLR	530.935	3	24.5
B2M	Beta-2-Microglobulin	SNFLNCYVSGFHPSDIEVDLLK	852.0810	3	33.0
B2M		VEHSDLSFSK	574.783	2	15.4
C3	Complement C3	TIYTPGSTVLYR	685.869	2	24.5
C4BPA	C4b-binding protein alpha chain	EDVYVVGTVLR	625.343	2	27.5
C4BPA		FSAICQGDGTWSPR	791.359	2	22.6
CAMP	Cathelicidin Antimicrobial Peptide	FALLGDFFR	543.293	2	34.6
CAMP		SSDANLYR	463.2220	2	13.3
CLU	Clusterin	ELDESLQVAER	644.8230	2	19.7
CST3	Cystatin C	ALDFAVGEYNK	613.8060	2	24.5
GC	Vitamin D-binding protein	SCESNSPFPVHPGTAECCTK	755.6510	3	18.5
GC		VCSQYAAYGEK	638.287	2	14.5
HPR	Haptoglobin-related protein	NYAEVGR	404.701	2	13.3
HPR		VGYVSGWGQSDNFK	772.3620	2	23.6
IGF1	Insulin-like growth factor I	RAPQTGIVDECCFR	570.263	3	18.5

IGF2	Insulin-like growth factor II	GIVEECCFR	585.258	2	18.8
IGF2		SCDLALLETYCATPAK	906.929	2	28.2
IHH	Indian hedgehog protein	AFQVIETQDPPR	700.862	2	21.6
IHH		ATFASHVQPGQYVLVAGVPGLQPAR	855.131	3	30.7
ITGB1	Integrin beta-1	IGFGSFVEK	492.264	2	26.2
ITIH4	Inter-alpha-trypsin inhibitor heavy chain H4	ILDDLSPR	464.759	2	20.4
ITIH4		IPKPEASFSPR	410.228	2	17.1
LCAT	Lecithin:cholesterol acyltransferase	SSGLVSNAPGVQIR	692.8810	2	21.6
LCAT		STELCGLWQGR	653.8140	2	25.3
LPA	Apolipoprotein(a)	GTYSTTVTGR	521.7620	2	13.4
LPA		NPDAVAAPYCYTR	749.3430	2	19.0
MENT	Methylated in normal thymocytes protein	SSAINEEDGSSEEGVVINAGK	1046.488	2	19.6
MENT		VGALSQLR	422.256	2	17.7
ORM1	Alpha-1-acid glycoprotein 1	EQLGEFYEALDCLR	871.906	2	32.2
ORM2	Alpha-1-acid glycoprotein 2	EQLGEFYEALDCLCIPR	1056.99	2	34.2
PCYOX1	Prenylcysteine oxidase 1	TLLETLQK	473.284	2	22.1
PLA2G7	Platelet-activating factor acetylhydrolase	GSVHQNFADFTFATGK	576.278	3	25.5
PLTP	Phospholipid transfer protein	ATYFGSIVLLSPAVIDSPLK	1046.09	2	35.0

PLTP		AVEPQLQEEER	664.328	2	15.7
PON1	Paraoxonase/arylesterase 1	IQNILTEEPK	592.8300	2	21.3
PON1		SFNPNSPGK	474.233	2	13.3
RBP4	Retinol Binding Protein 4	YWGVASFLQK	599.8160	2	31.3
SAA1	Serum Amyloid A1	FFGHGAEDSLADQAANEWGR	726.6590	3	25.6
SCGB3A1	Secretoglobin family 3A member 1	CVAELGPQAVGAVK	699.874	2	22.0
SCGB3A1		LLLSSLGIPVNHLIEGSQK	1009.586	2	32.5
SCGB3A1		LLLSSLGIPVNHLIEGSQK	673.393	3	32.5
SELL	L-selectin	SYYWIGIR	529.277	2	28.9
SERPINA1	Alpha-1-antitrypsin	LSITGTYDLK	555.8060	2	23.8
SERPINA1		SVLGQLGITK	508.3110	2	26.2
SFTPB	Pulmonary surfactant-associated protein B	SPTGEWLPR	521.769	2	22.0
SFTPB		YSVILLDTLLGR	681.903	2	34.9
TTY	Transthyretin	AADDTWEPFASGK	697.8148	2	24.5
TTY		TSESGELHGLTTEEEFVEGIYK	819.055	3	30.0
VTN	Vitronectin	DVWGIEGPIDAAFTR	823.912	2	33.5
VTN		FEDGVLDPDYPR	711.83	2	24.5

Supplemental Table 2. PRM analysis of HDL proteins in T1DM patients with or without CVD events in CACTI.

Proteins	Description	Control	CVD	P-value	Q-value
**SFTPB**	**Pulmonary surfactant-associated protein B**	**1±1.57**	**2.70±3.44**	**1.9E-05**	**0.00097**
**AMBP**	**Alpha-1-Microglobulin/Bikunin Precursor**	**1±0.87**	**1.46±1.15**	**0.00016**	**0.0041**
**IGF1**	**Insulin-like growth factor I**	**1±0.59**	**0.71±0.69**	**0.00045**	**0.0077**
**C4BPA**	**C4b-binding protein alpha chain**	**1±1.07**	**1.23±1.02**	**0.0030**	**0.038**
**CST3**	**Cystatin C**	**1±0.68**	**1.34±0.94**	**0.0089**	**0.091**
APOH	Beta-2-glycoprotein 1	1±0.63	1.48±1.43	0.021	0.17
B2M	Beta-2-Microglobulin	1±1.08	1.29±1.01	0.024	0.17
PTGDS	Prostaglandin-H2 D-isomerase	1±2.42	1.88±3.49	0.030	0.18
IGF2	Insulin-like growth factor II	1±0.62	0.86±0.72	0.031	0.18
VTN	Vitronectin	1±0.47	0.89±0.49	0.046	0.23
ApoA4	Apolipoprotein A-IV	1±0.52	1.16±0.71	0.049	0.23
ApoB	Apolipoprotein B	1±0.65	1.16±0.74	0.066	0.28
C3	Complement C3	1±0.47	1.13±0.54	0.079	0.3
ApoC3	Apolipoprotein C-III	1±0.54	1.27±1.02	0.082	0.3
SAA1	Serum Amyloid A1	1±2.11	1.02±1.14	0.11	0.37
RBP4	Retinol Binding Protein 4	1±0.50	1.25±0.85	0.14	0.42
ApoC2	Apolipoprotein C-II	1±0.57	1.14±0.70	0.14	0.42
MENT	Methylated in normal thymocytes protein	1±0.34	0.95±0.32	0.18	0.51
ORM1	Alpha-1-acid glycoprotein 1	1±0.54	1.05±0.43	0.22	0.58
ITGB1	Integrin beta-1	1±0.55	0.87±0.46	0.24	0.58
A2M	Alpha-2-macroglobulin	1±1.11	1.05±0.77	0.25	0.58
APOD	Apolipoprotein D	1±0.33	1.07±0.40	0.25	0.58

ANTXR2	Anthrax toxin receptor 2	1±0.38	1.06±0.39	0.30	0.66
PON1	Paraoxonase/arylesterase 1	1±0.35	0.96±0.37	0.33	0.67
AHSG	Alpha-2-HS-glycoprotein	1±0.35	0.96±0.36	0.36	0.68
IHH	Indian hedgehog protein	1±0.42	0.95±0.40	0.38	0.68
APOL1	Apolipoprotein L-I	1±0.47	0.94±0.48	0.38	0.68
GC	Vitamin D-binding protein	1±0.42	0.94±0.39	0.39	0.68
ApoE	Apolipoprotein E	1±0.47	0.95±0.53	0.42	0.68
CAMP	Cathelicidin Antimicrobial Peptide	1±0.40	1.04±0.38	0.43	0.68
ApoC1	Apolipoprotein C-I	1±0.32	1.06±0.41	0.44	0.68
SCGB3A1	Secretoglobin family 3A member 1	1±0.75	0.98±0.48	0.44	0.68
ApoA2	Apolipoprotein A-II	1±0.23	1.06±0.33	0.48	0.71
ApoA1	Apolipoprotein A-I	1±0.30	1.04±0.34	0.49	0.71
ApoC4	Apolipoprotein C-IV	1±0.53	1.06±0.56	0.50	0.71
CLU	Clusterin	1±0.43	0.93±0.34	0.54	0.73
ORM2	Alpha-1-acid glycoprotein 2	1±0.49	1.00±0.63	0.55	0.73
LPA	Apolipoprotein(a)	1±1.47	1.02±1.25	0.57	0.73
PCYOX1	Prenylcysteine oxidase 1	1±0.36	1.03±0.41	0.58	0.73
PLA2G7	Platelet-activating factor acetylhydrolase	1±0.40	0.96±0.44	0.59	0.73
PLTP	Phospholipid transfer protein	1±0.45	0.96±0.37	0.61	0.74
APOM	Apolipoprotein M	1±0.40	1.01±0.43	0.66	0.78
ITIH4	Inter-alpha-trypsin inhibitor heavy chain H4	1±0.89	1.09±1.23	0.71	0.82
LCAT	Lecithin:cholesterol acyltransferase	1±0.37	1.01±0.43	0.75	0.85
SERPINA1	Alpha-1-antitrypsin	1±1.05	1.12±1.22	0.81	0.9
SELL	L-selectin	1±0.53	1.00±0.44	0.89	0.97
TTY	Transthyretin	1±0.33	1.07±0.65	0.93	0.99

ANTXR1	Anthrax toxin receptor 1	1±0.43	0.98±0.37	0.95	0.99
HPR	Haptoglobin-related protein	1±0.63	1.14±0.93	0.97	0.99
ApoF	Apolipoprotein F	1±0.34	1.01±0.35	0.99	0.99

The number of subjects was 181. There were 145 subjects in the cohort group (including 11 CVD subjects). HDL was isolated from serum. Following digestion of HDL with trypsin, HDL proteins were analyzed by isotope dilution targeted MS/MS with parallel reaction monitoring (PRM). The level of each HDL protein from 134 cohort subjects without CVD events was defined as 1.00. Data are means ± SDs. Because the levels of proteins in HDL are not normally distributed, P values were obtained by a Mann-Whitney non-parametric test. The proteins significantly associated with incident CVD after controlling the Benjamini-Hochberg false discovery rate at 10% (FDR adjusted P-values or Q-values < 0.10) are shown in bold.

Supplemental Table 3. Correlations between levels of SFTPB in HDL and other HDL proteins.

Protein	Correlation	*P*-value		Protein	Correlation	*P*-value
APOC3	0.337	3.4E-06		APOC4	0.226	0.0022
APOM	0.325	7.9E-06		CST3	0.224	0.0024
SCGB3A1	0.325	8.2E-06		ApoA2	0.222	0.0027
IGF1	−0.321	1.0E-05		AMBP	0.218	0.0032
C4BPA	0.315	1.6E-05		SAA1	0.212	0.0043
SELL	0.302	3.6E-05		ANTXR2	0.209	0.0048
APOD	0.299	4.3E-05		PCYOX1	0.2	0.0069
APOC2	0.263	0.00034		HPR	0.198	0.0076
RBP4	0.242	0.0010		APOA4	0.182	0.014
APOH	0.239	0.0012		IHH	0.18	0.016
APOA1	0.237	0.0013		APOB	0.17	0.022
APOC1	0.23	0.0018		A2M	0.167	0.025

The number of subjects was 181. There were 145 subjects in the cohort group (including 11 CVD subjects) and additional 36 incident CVD subjects outside of the cohort group. HDL was isolated from serum. Following digestion of HDL with trypsin, HDL proteins were analyzed by isotope dilution targeted MS/MS with parallel reaction monitoring (PRM). Unadjusted Pearson correlation analysis was performed between the levels of SFTPB and all other HDL proteins. The 24 proteins that were significantly associated with SFTPB ($P<0.05$) are listed. See Supplemental Table 2 for protein names.

Supplemental Table 4. Hazard Ratios of HDL Proteins for Incident CVD.

Models	Hazards Ratio	95% CI		*P*-value	Q-value
**SFTPB**	**2.22**	**1.54**	**3.19**	**1.7E-05**	**0.00088**
**IGF1**	**0.57**	**0.41**	**0.80**	**0.0010**	**0.026**
**AMBP**	**1.66**	**1.19**	**2.30**	**0.0026**	**0.045**
CST3	1.52	1.11	2.08	0.0085	0.11
APOH	1.66	1.12	2.46	0.011	0.12
PTGDS	1.48	1.04	2.10	0.028	0.20
C4BPA	1.38	1.03	1.85	0.029	0.20
B2M	1.40	1.03	1.89	0.031	0.20
APOC3	1.40	1.00	1.95	0.048	0.27
VTN	0.74	0.53	1.03	0.077	0.39
C3	1.32	0.96	1.82	0.086	0.39
APOB	1.30	0.95	1.77	0.097	0.39
APOA4	1.29	0.95	1.75	0.10	0.39
IGF2	0.79	0.60	1.05	0.11	0.40
RBP4	1.33	0.92	1.93	0.13	0.43
APOC2	1.25	0.92	1.72	0.16	0.50
SAA1	1.24	0.91	1.69	0.17	0.52
APOL1	0.80	0.57	1.12	0.20	0.57
ITGB1	0.83	0.62	1.13	0.24	0.60
APOA2	1.21	0.88	1.66	0.24	0.60
APOD	1.21	0.88	1.66	0.25	0.60
PLA2G7	0.83	0.60	1.16	0.28	0.61
ORM1	1.19	0.86	1.66	0.30	0.61

ANTXR2	1.19	0.86	1.63	0.30	0.61
PON1	0.85	0.61	1.18	0.33	0.65
A2M	1.16	0.85	1.58	0.34	0.65
CLU	0.87	0.63	1.19	0.38	0.67
AHSG	0.86	0.62	1.20	0.38	0.67
MENT	0.87	0.63	1.20	0.39	0.67
IHH	0.88	0.65	1.19	0.41	0.67
APOC1	1.14	0.82	1.59	0.44	0.68
SCGB3A1	1.13	0.82	1.55	0.45	0.68
APOA1	1.13	0.82	1.56	0.47	0.68
HPR	1.12	0.81	1.57	0.49	0.68
APOC4	1.11	0.81	1.52	0.51	0.68
ORM2	0.90	0.67	1.22	0.51	0.68
SERPINA1	1.11	0.81	1.52	0.52	0.68
APOE	0.90	0.65	1.25	0.53	0.68
CAMP	1.11	0.81	1.52	0.54	0.68
SELL	1.09	0.80	1.50	0.57	0.71
PCYOX1	1.09	0.79	1.51	0.60	0.72
GC	0.93	0.69	1.24	0.61	0.72
PLTP	0.94	0.69	1.29	0.71	0.82
TTY	1.05	0.74	1.49	0.77	0.87
LCAT	0.97	0.70	1.34	0.83	0.91
ANTXR1	0.97	0.70	1.33	0.84	0.91
APOM	1.03	0.74	1.42	0.87	0.91
APOF	1.03	0.74	1.42	0.88	0.91

ITIH4	0.98	0.70	1.37	0.90	0.92
LPA	1.01	0.73	1.40	0.96	0.96

Unadjusted hazard ratios, 95% confidence interval (CI) and P values are from Cox proportional hazard ratio models using a case-cohort design and the "cch" function in R with the default Prentice weighting. The total number of subjects included was 181 and there were 145 randomly selected subjects in the cohort. There were 47 subjects with CVD events and the cohort contained 11 incident CVD subjects. Hazard ratios are per SD increase of levels of HDL proteins. Proteins significantly associated with incident CVD after controlling the Benjamini-Hochberg false discovery rate at 10% (FDR adjusted P-values or Q-values < 0.1) are shown in bold. See Supplemental Table 2 for protein names.
