# Supplementary material for: Pulmonary surfactant protein B carried by HDL predicts incident CVD in patients with type 1 diabetes
Source: J Lipid Res. 2022 Mar 14;63(4):100196. doi: 10.1016/j.jlr.2022.100196 (PMC9010748; doi:10.1016/j.jlr.2022.100196)
Supplement: Supplemental Tables S1–S2 [file mmc2.pdf]

Supplemental Figures

Supplemental Figure 1.

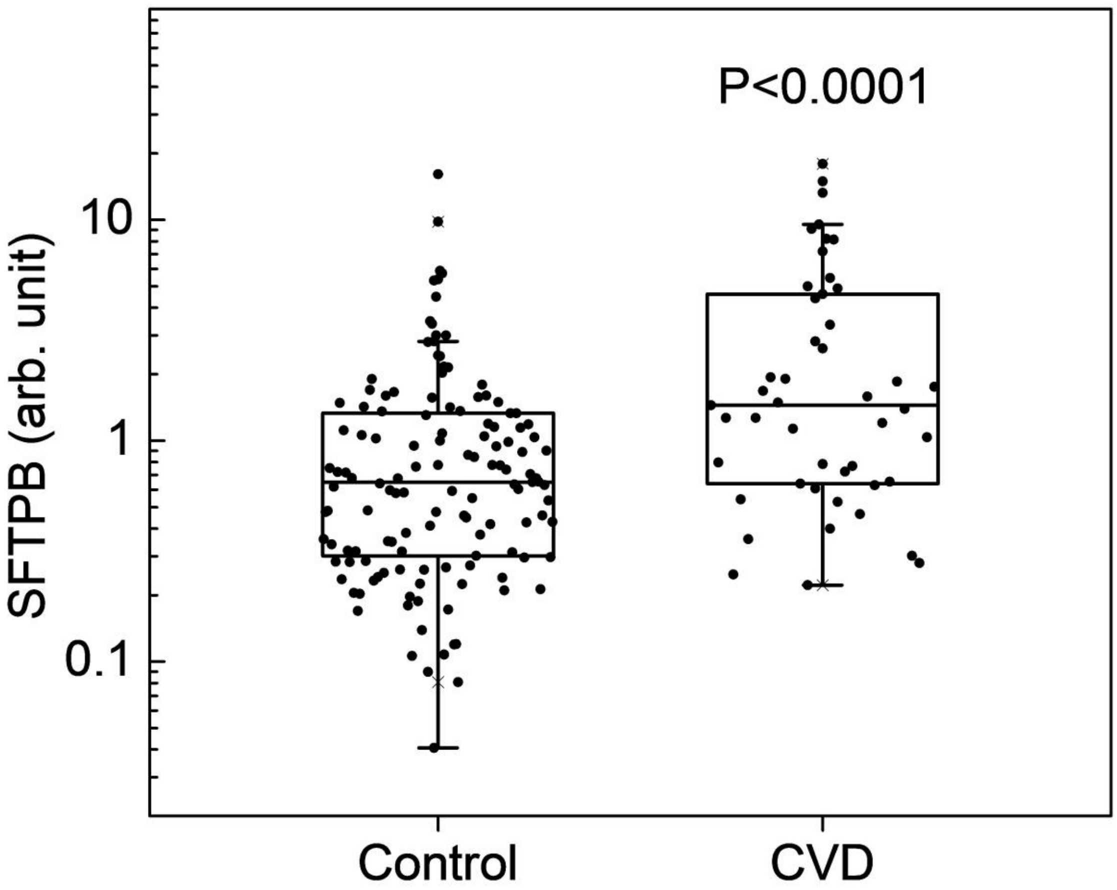

Levels of SFTPB in HDL of incident CVD subjects (n=47, including 11 in the cohort group) and control subjects (cohort subjects without incident CVD, n=134). Levels of SFTBP in HDL were quantified by isotope dilution PRM. The mean level of SFTPB in control subjects (cohort subjects without incident CVD) was defined as 1.00. SFTBP levels are logarithmically transformed because they were not normally distributed (Shapiro-Wilk test). Box plots show the distribution of the levels of SFTPB in HDL (median, interquartile ranges); dots represent individual data points. P values are from a Mann-Whitney U test. SFTPB, pulmonary surfactant protein B.

Supplemental Figure 2.

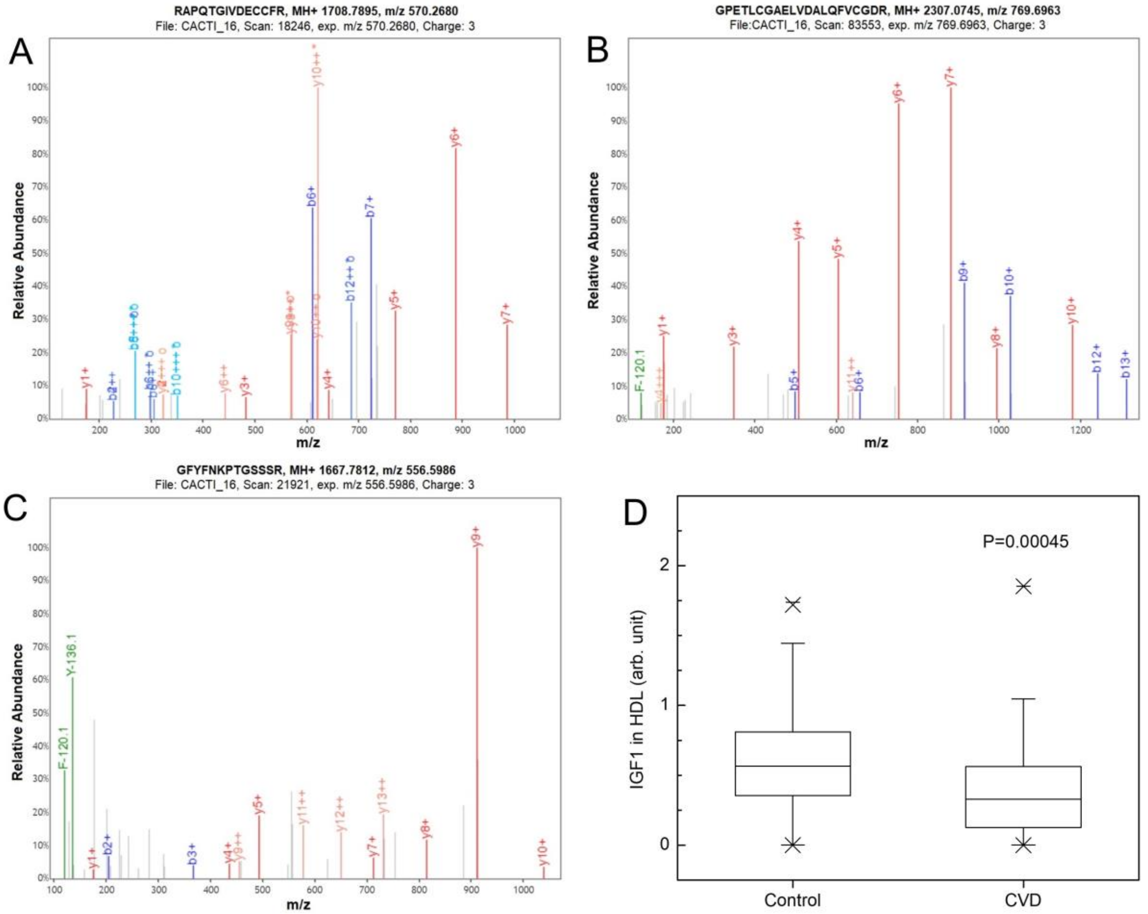

Identification and quantification of IGF1 in HDL. (**A-C**) Detection of 3 unique peptides of IGF1 by LC-ESI-MS/MS in tryptic digests of HDL. (**D**) Quantification of IGF1 in tryptic digests of HDL by isotope dilution PRM analysis. Box plots show the distribution of the levels of IGF1 in HDL (median, interquartile ranges). P value is from Mann-Whitney U test. IGF1, insulin-like growth factor I.

Supplemental Figure 3.

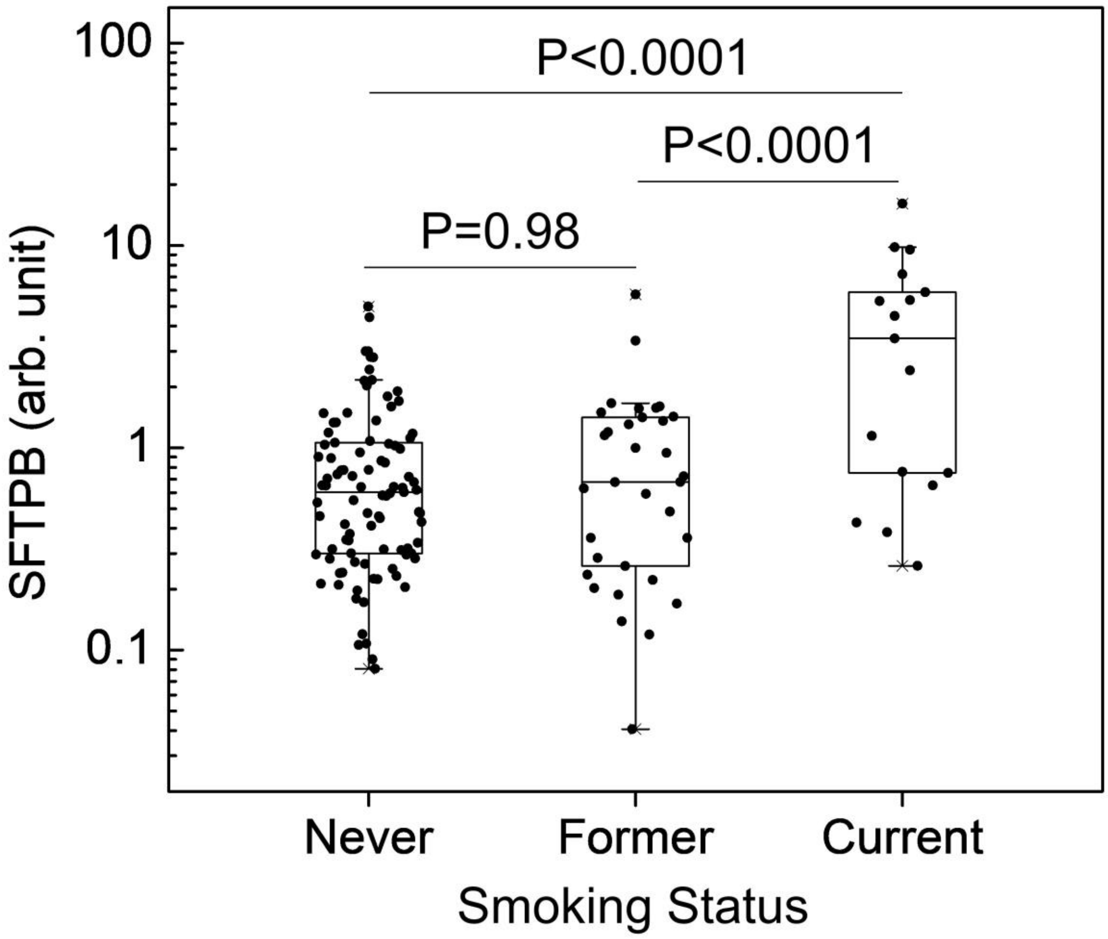

Levels of SFTPB in HDL of current smokers (n=17), former smokers (n=33) and subjects that never smoked (n=95) within the cohort group. Levels of SFTBP in HDL were quantified by isotope dilution PRM. The mean level of SFTPB in control subjects (cohort subjects without incident CVD) was defined as 1.00. SFTBP levels are logarithmically transformed because they were not normally distributed (Shapiro-Wilk test). Box plots show the distribution of the levels of SFTPB in HDL (median, interquartile ranges); dots represent individual data points. P values are from One-way ANOVA post hoc Tukey HSD test. SFTPB, pulmonary surfactant protein B.

Supplemental Figure 4.

Pro-Surfactant Protein B: Length, 381; Mass (Da), 42,117

MAESHLLQWL LLLLPTLCGP GTAAWTTSSL ACAQGPEFWC
QSLEQALQCR ALGHCLQEVW GHVGADDLCQ ECEDIVHILN
KMAK EAIFQDTMR K FLEQECNVLPLK LLMP QCNQVLDDYF
PLVIDYFQNQ TDSNGICMHL GLCKSRQPEP EQEPGMSDPL
PKPLRDPLPD PLLDKLVLPV LPGALQARPG PHTQDLSEQQ
FPIPLPYCWL CRALIKRIQA MIPK GALAVAVAQVCR VVPL
VAGGICQCLAER YSVILLDTLLGR MLPQLVCR LVLR **CSM**
DDSAGPR SPTGEWLPR DSECH LCMS VTTQAG
NSSEQAIPQA MLQACVGSWL DREKCK QFVEQHTPQLLTLV
PR GWDAHTTC QALGVCGTMS SPLQCIHSPD L

Mature Surfactant Protein B: Length, 79; Mass (Da), 8,704

FPIPLPYCWLCRALIKRIQAMIPK GALAVAVAQVCR VVPL
VAGGICQCLAER YSVILLDTLLGR MLPQLVCR LVLRCSM

Sequence of pro- and mature SFTPB. Mature SFTPB is shown in blue. Tryptic peptides from SFTPB detected by mass spectrometry in HDL isolated from CACTI subjects are underlined. Peptide **CSMDDSAGPR** is present in both mature SFTPB and in the propeptide domains of the protein.

Supplemental Figure 5.

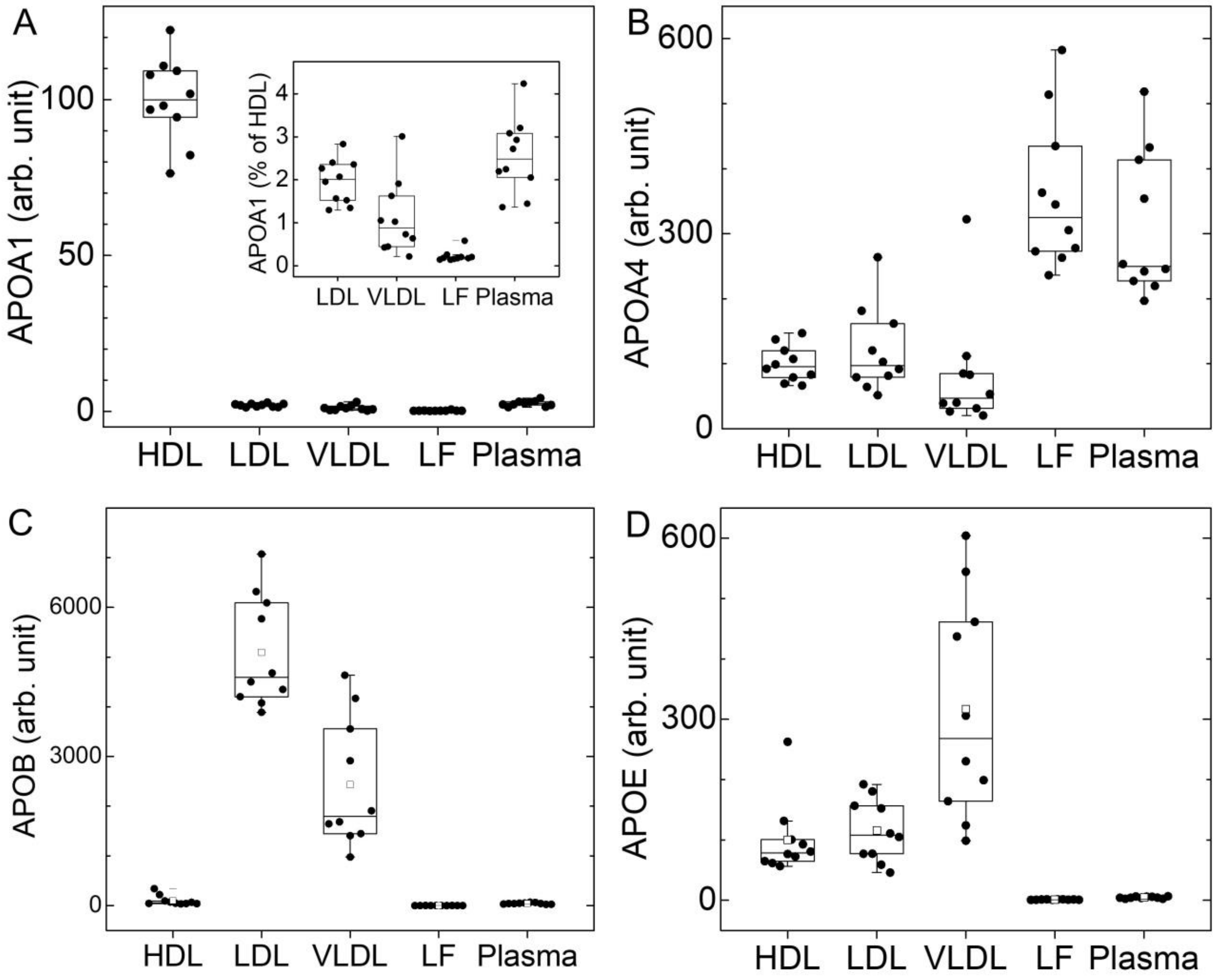

Association of apolipoproteins with different lipoprotein fractions isolated from plasma. HDL, LDL, VLDL, lipoprotein-free plasma (LF) were isolated by ultracentrifugation of plasma. Levels of (A) APOA1, (B) APOA4, (C) APOB, and (D) APOE were quantified by isotope dilution PRM. Mean levels of the apolipoproteins in HDL were defined as 100. Relative levels of apolipoproteins in other lipoproteins and lipoprotein-free plasma are expressed as percent of the levels in HDL. Data are from plasma of 10 CACTI subjects with T1DM. Panel A is the same APOA1 data as panel B of Figure 4 in the paper. We used the data in Figure 4 to compare the distribution of APOA1 and SFTPB in plasma. Here we use the data to compare the levels of APOA1 with that of other apolipoproteins (APOB, APOE, APOA4) in HDL, LDL, VLDL, and lipoprotein-free plasma.

Supplemental Figure 6.

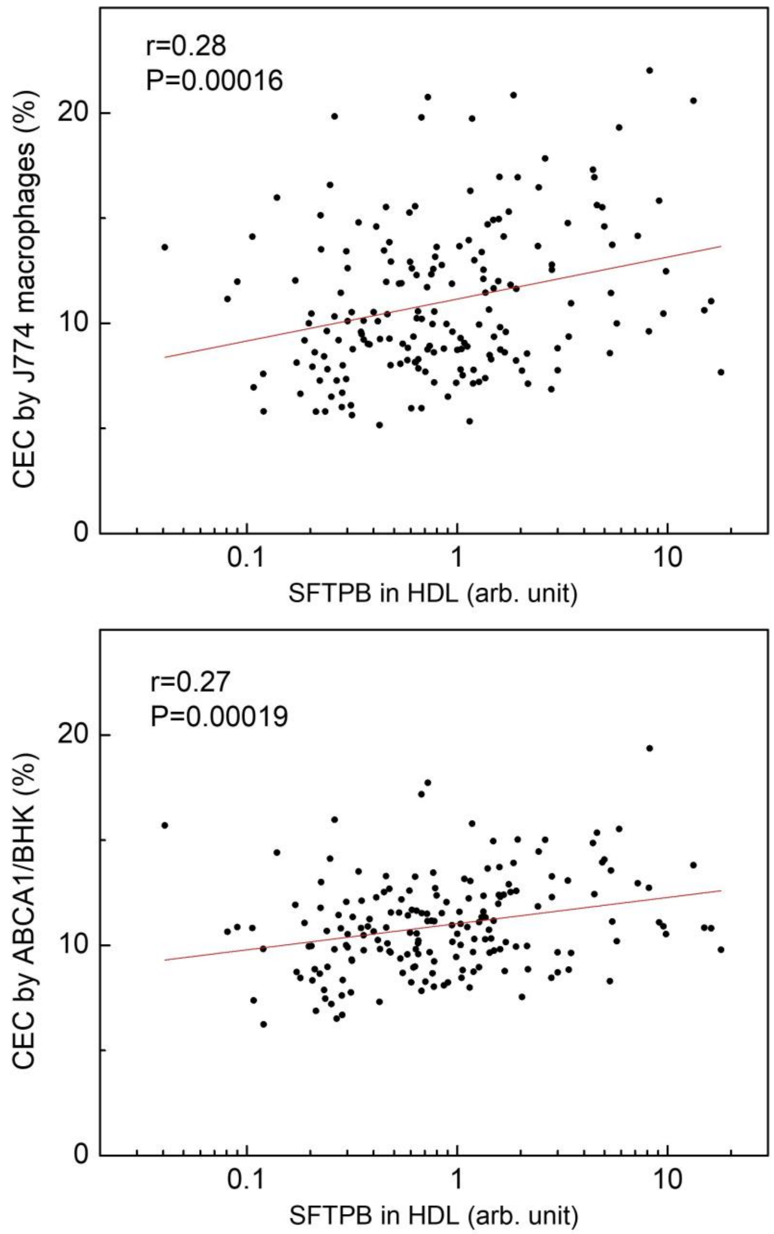

Association of serum HDL's cholesterol efflux capacity (CEC) and levels of SFTPB in HDL. Cholesterol efflux capacity of serum HDL was measured as described in the Methods section. The levels of SFTPB in HDL were measured by isotope dilution targeted MS/MS with PRM. Because the levels of SFTPB in HDL are not normally distributed, the data was log transformed and then Pearson correlation between levels of SFTPB in HDL and CEC was performed.

Supplemental Figure 7.

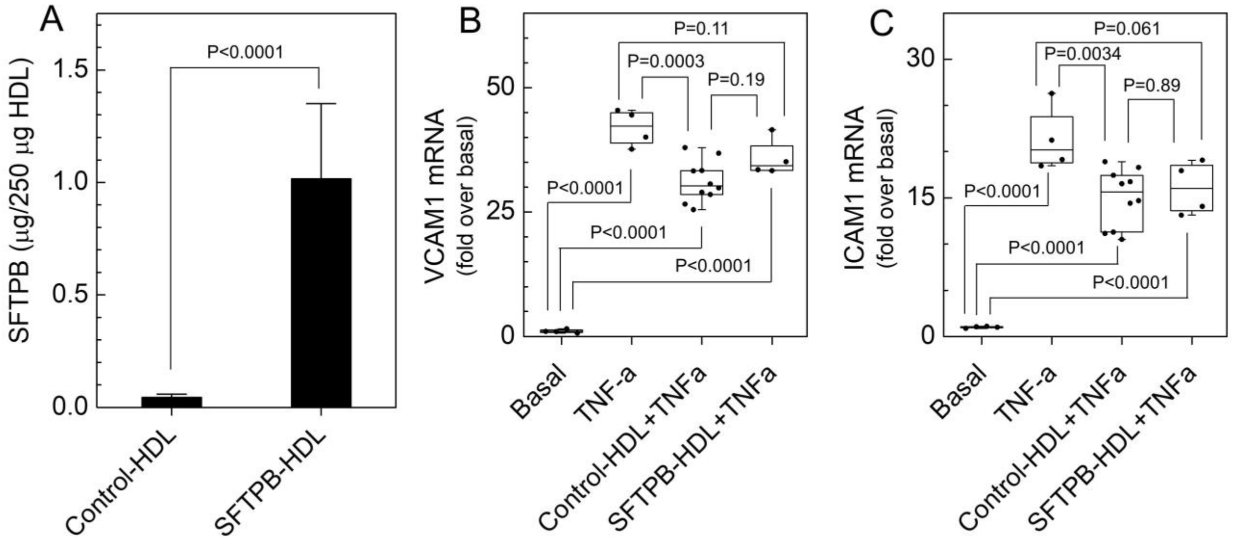

Enrichment of SFTPB in HDL (A) and effect of SFTPB-enriched HDL on adhesion molecule gene expression in TNF-α-stimulated human coronary endothelial cells (B and C). (A) Control-HDL was incubated with pro-SFTPB, SFTPB-enriched HDL was reisolated by ultracentrifugation and the levels of SFTPB was measured by PRM analysis. Results are means with standard deviations (n=3 technical replicates and representative experiment among 2 independent experiments are shown). P-values are from Student's t-test. (B and C) Human coronary endothelial cells were pre-treated with control-HDL or SFTPB-enriched HDL (50 µg/mL), then stimulated with TNF-α (20 ng/ml) for an additional 6 hr. VCAM1 and ICAM1 mRNA levels were evaluated by real time PCR. The box plots show the distribution of the levels of VCAM1 or ICAM1 (median, interquartile ranges), while the dots represent individual data points. P-values are from One-way ANOVA followed by Tukey's multiple comparison test.
